# Failure of an Educational Intervention to Improve Consultation and Implications for Healthcare Consultation

**DOI:** 10.7759/cureus.4105

**Published:** 2019-02-20

**Authors:** Joseph Turner, Megan Litzau, Zachary S Morgan, Katherine Pollard, Dylan D Cooper

**Affiliations:** 1 Emergency Medicine, Indiana University School of Medicine, Indianapolis, USA; 2 Emergency Medicine, Mercy Hospital, St. Louis, USA

**Keywords:** consultation skills, medical student education

## Abstract

Introduction: Consultation of another physician for his or her specialized expertise regarding a patient’s care is a common occurrence in most physicians’ daily practice, especially in the emergency department (ED). Therefore, the ability to communicate effectively with another physician during a patient consultation is an essential skill. However, there has been limited research on a standardized method for a physician to physician consultation with little guidance on teaching consultations to physicians in training. The objective of our study was to measure the effect of a structured consultation intervention on both content standardization and quality of medical student consultations.

Methods: Senior medical students were assessed on a required emergency medicine rotation with a physician phone consultation during a standardized, simulated chest pain case. The intervention groups received a standard consult checklist as part of their orientation to the rotation, followed by a video recording of a good consult call and a bad consult call with commentary from an emergency physician. The intervention was given to students every other month, alternating with a control group who received no additional education. Recordings were reviewed by three second-year internal medicine residents pursuing a fellowship in cardiology. Each recording was evaluated by two of the three reviewers and scored using a standardized checklist.

Results: Providing a standardized consultation intervention did not improve students’ ability to communicate with consultants. In addition, there was variability between evaluators in regards to how they received the same information and how they perceived the quality of the same recorded consultation calls. Evaluator inter-rater reliability (IRR) was poor on the questions of 1) would you have any other questions of the student calling the consult and 2) did the student calling the consult provide an accurate account of information and case detail. The IRR was also poor on objective data such as whether the student stated their name.

Conclusions: A brief intervention may not be enough to change complex behavior such as a physician to physician consultant communication. Importantly, despite consultants listening to the same audio recordings, the information was processed differently. Future investigations should focus on both those delivering as well as those receiving a consultation.

## Introduction

Consultation of another physician for his or her specialized expertise regarding a patient’s care is a common activity in a physician’s daily life, but is especially common in the emergency department (ED). A recent study by Lee et al. demonstrated that at least 20% of patients admitted from the ED receive at least one consultation regarding their care [1]. The ability to communicate effectively to another physician during this consultation process is an essential skill for any physician.

Despite the impact on patient care and the frequency of these consultations in the practice of medicine, there has been limited research regarding the best method to teach this skill to providers in training. The five Cs consultation model: contact, communication, core question, collaboration, and closing the loop series was developed and studied which did show improvement in the consultation skills of physicians [2-4]. Additional studies recommended focusing on organizational skills, interpersonal and communication skills, and medical knowledge when teaching consultation skills [5]. Our study was based on an institutional effort to improve communication in consultations and at our institution, the five Cs was not being used as the basis of this effort.

The objective of our study was to measure the effect of a new structured intervention at standardizing the content of medical student consultations and to assess the impact of the intervention on the quality of the consultation as perceived by the receiving physician.

## Materials and methods

This was a prospective, interventional study conducted at the Simulation Center at Fairbanks Hall in association with the Indiana University School of Medicine, Department of Emergency Medicine. Eligible participants included all senior medical students on a required fourth-year emergency medicine clerkship over a 16 month period. Students participated in a mandatory simulation session as part of their clerkship requirements and were informed that the sessions would be used for research purposes, but were blinded to the nature of the study. The study was approved by the Institutional Review Board and was assigned protocol number 1405001411A001.

The students managed a mannequin-based case of a patient with chest pain who required cardiology consultation. Two students cared for the patient as a team, with one of those two students per group consulting a cardiologist over the phone. The cardiologist was played by an emergency medicine faculty using a standardized script. These consultation calls were audio recorded.

**Figure 1:**
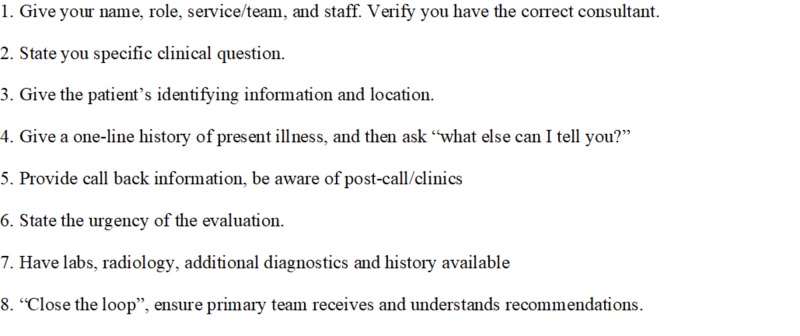
Standard Consult Format

The Graduate Medical Education Committee at Indiana University School of Medicine developed a “consultation card”, with plans to pilot the card before an institution-wide rollout. For the educational intervention, students were given the consultation card in their clerkship orientation packet and they were encouraged to use it during all consultations throughout the rotation (Figure 1). Students in the intervention group also viewed a video recording of a good consult call and a bad consult call, with the good consult call based on the standardized checklist. An emergency physician provided commentary about the quality of each consultation. The intervention was offered during alternating months with the other students receiving no formal education and serving as the control group.

The audio recordings of the intervention group and the control group were then evaluated by three male second-year internal medicine residents who are all applying for cardiology fellowship. Evaluators were asked to score the content and quality of each call using a standard form based on the checklist (Tables 1-2) They were kept blind to group assignment. Each call was evaluated by two of the three evaluators. Inter-rater reliability (IRR) for the nominal items (with response options of “no”, “yes”, or “yes - with prompting”) was measured between each of these pairs of raters using Cohen’s kappa. IRR for the ordinal items (“not effective” to “very effective” on a four-point scale) was calculated using the single measure values from one-way random intra-class correlations.

**Table 1:** Consultant Objective Interaction Checklist

	Performed	Not Performed	Comments
States name			
States rank and service			
Identifies supervising attending			
Identifies name of consultant physician			
Presents a concise story			
Presents an accurate recount of information/case detail			
Speaks clearly			
Specifies need for consultation			
Specifies timeframe for consultation			
Is open to and incorporates consultant’s recommendations			
Reviews and repeats patient care plan			
Thanks consultant for consultation			
TOTAL			

**Table 2:** Consultant Subjective Interaction Checklist

	1 Not effective	2 Somewhat effective	3 Effective	4 Very effective	5 Extremely effective
Introduction of involved parties					
Specified consultation objective					
Patient case presentation					
Case discussion					
Confirmation and closing					
Interpersonal skills					
Global rating					

To measure the effectiveness of the intervention, chi-square tests were run on the nominal items and independent sample t-tests were run on the ordinal items.

## Results

Overall, 70 recordings were evaluated – 23 from students who had received the intervention and 47 from students who had not. There were fewer students in the intervention group as the intervention was not provided in four months during the project course.

IRR results for the nominal can be found in Table 3. Evaluators A and B were the most in agreement, with perfect agreement on three items and values above the 0.6 threshold on another four items. Evaluators B and C were in perfect agreement on one item and above the threshold for only one other; Evaluators A and C did not even meet the 0.6 threshold for a single item. The IRR for the ordinal items, available in Table 4, found little agreement, with no pair of raters meeting a 0.6 threshold for more than three of the seven items.

**Table 3:** Cohen’s Kappa Measures of Inter-rater Reliability on Nominal Evaluation Items

Item	A with B	A with C	B with C
States name (replaced with beep in recording)	.912***	.374*	.751***
States role	.838***	.209*	.143
Identifies name of consultant	.832***	.378*	1.000***
Presents a concise story	.302*	.238	.472**
Presents an accurate recount of information/case detail	.012	.086	.046
Speaks clearly	***	ERR	ERR
Specifies need for consultation	.708***	.083	-.092
Specifies time frame for consultation	.160*	.271*	-.259*
Is open to and incorporates consultant's recommendations	***	ERR	ERR
Reviews and repeats patient care plan	.250	.176	-.185
Thanks consultant for consultation	ERR	ERR	.233
Would you accept this patient?	***	ERR	-.045
Would you have any other questions?	-.067	ERR	-.062
*p			

**Table 4:** Intra-class Correlation Coefficients for Ordinal Evaluation Items

	A with B	A with C	B with C
Introduction of involved parties	0.432*	0.611***	0.423
Specified consultation objective	0.744***	-0.231	0.188
Patient case presentation	0.396*	0.627***	0.809***
Case discussion	-0.043	0.152	0.509**
Confirmation and closing	0.098	0.299	0.157
Interpersonal skills	-0.185	0.189	0.374*
Global rating	0.085	0.632***	0.610***

Results of the chi-square analysis on the nominal items found no significant differences between the control or intervention group, nor did the independent sample t-tests find significant differences between the means of the ordinal measures of effectiveness (Table 5).

**Table 5:** Independent Sample T-tests for Ordinal Evaluation Items

Item	Control			Intervention		
	N	M	SD	N	M	SD
Introduction of involved parties	94	2.18	0.93	47	2.00	0.91
Specified consultation objective	94	2.32	0.79	47	2.13	0.90
Patient case presentation	94	1.95	0.82	47	1.98	0.92
Case discussion	94	2.14	0.65	47	2.00	0.72
Confirmation and closing	94	2.11	0.66	47	1.87	0.80
Interpersonal skills	94	2.31	0.73	47	2.04	0.83
Global rating	94	2.20	0.80	47	2.02	0.92

Evaluator ratings of each of the seven outcomes measures of consultation effectiveness can be found in Table 4. There were no significant differences between the intervention group and the control group for any of the seven outcome measures.

## Discussion

In this study, we attempted to improve medical student consultation using a structured educational intervention. We did not find a benefit in any of the measured outcomes from our intervention. This may indicate that our intervention was not robust enough to influence complex behavior like communication with a consultant.

However, our data also indicate that assessment of interpersonal interactions such as consultation must be viewed with caution. In our study, we found substantial disagreement in the assessment between different reviewers. One might expect significant disagreement for questions that have a subjective component or that require interpretation. However, we found that there was frequent disagreement even for objective questions such as whether the student stated their name or whether they stated their role.

Our intervention was targeted to provide a standardized format for our students to convey their patient’s information to the consultant. While we standardized the intervention, simulated patient case and the consultant interacting with the student on the recording, we could not standardize the individual evaluators who reviewed the recordings and their individual preferences. Our question was focused solely on an intervention for the provider calling the consult. We did not account for the variability in how consultants receive information. Despite our evaluators listening to the same audio recordings, they were receiving the information differently. As such, this makes it difficult to provide students instruction on what their consultants require in the consultation call when there is disagreement amongst the receivers of the information. We hypothesized the reason for this finding could be based on individual practice of the provider, the order of preferred information by the receiver of the information, how the receiver of the information was processing the information and how the provider calling the consultation stated the information.

As noted above, the students in the intervention group required more prompting on the basic elements of a consultation call including stating their name and role. This was found in contrast to a study by Go et al. [6] in which they were able to train medical students to state their name and department. We hypothesized that one possible reason behind our intervention being less successful in this area was that our intervention was disrupting how the student would have naturally called the consultant which would have included an introduction on their own. In addition, students did not have the consult checklist in front of them when actually placing their consultation calls during the simulation. As such, we hypothesized that the intervention students may have been trying to think ahead and remember the specific order and components of the call and by doing so, missed including some of the most basic information in the call. In order to answer this question, further research would need to be done.

Though our study did not show that the intervention had benefit for students, it did open more questions regarding the critical aspect of provider communication. This is relevant to all in medicine as we work to improve patient handover, patient safety and ensuring communication with other providers is as clear as possible. However, in the midst of trying to improve training and the recent movement in medicine towards standardization, there does remain an art to practicing medicine that we did not account for in our study. 

## Conclusions

Physician consultation involves the transfer of information and discussion between two people who may be entering the communication with different perspectives and expectations. Such a brief and focused intervention may not be enough to change this complex behavior. Despite our evaluators listening to the same audio recordings, they were processing the information differently. Future investigations should focus on both those delivering as well as those receiving a consultation.
